# LncRNA ZNF674-AS1 Hinders Proliferation and Invasion of Hepatic Carcinoma Cells through the Glycolysis Pathway

**DOI:** 10.1155/2022/8063382

**Published:** 2022-07-13

**Authors:** Dongliang Li, Yan Xie, Jisan Sun, Li Zhang, Wentao Jiang

**Affiliations:** ^1^Tianjin Medical University, First Central Clinical College, Tianjin 300192, China; ^2^Department of General Surgery, Lu'An Affiliated Hospital of Anhui Medical University, Lu'An, Anhui 237005, China; ^3^Department of Liver Transplantation, Tianjin First Central Hospital, Tianjin 300192, China

## Abstract

**Purpose:**

Long noncoding RNAs (lncRNAs) play important roles in regulating various functions of cells at the levels of transcription and posttranscription. Extensive investigations have illustrated that lncRNAs are critical in the glucose metabolism of tumor cells, but their mechanisms of action need to be further explored. This study evaluates the role of lncRNA ZNF674-AS1 on the apoptosis and proliferation of human hepatic carcinoma cells in vitro through the glucose metabolism and its related mechanisms.

**Methods:**

Real-time quantitative PCR was employed for detecting the level of expressions for lncRNA ZNF674-AS1 in liver cancer tissues (25 cases), paracancerous tissues, and liver cancer cell lines. The lncRNA ZNF674-AS1 high expression cell strain was constructed by the lentiviral overexpression vector. CCK-8, plate colony formation, transwell assay, lactate production, glucose consumption, and ATP levels were used to detect the change of cell proliferation, colony formation, migration, and invasion, as well as glycolytic capability. Western blot was carried out to detect the expression of HK2, PFKL, PKM2, GLUT1, and PKM1, which are the key proteins of glycolysis in cells.

**Result:**

The lncRNA ZNF674-AS1 was undesirably expressed in liver cancer cell lines and tissues. Cell function assessments showed that compared with the blank control group (vector), overexpression of lncRNA ZNF674-AS1 could substantially hinder the proliferation, colony formation, migration, and invasion capability of liver cancer cells. Furthermore, overexpression of lncRNA ZNF674-AS1 could inhibit cell glycolysis (inhibit glucose consumption and reduce intracellular lactate and ATP levels) by inhibiting the expression of key proteins (such as PFKL, HK2, PKM2, and GLUT1) in the process of glycolysis.

**Conclusion:**

As a tumor repressor gene, lncRNA ZNF674-AS1 inhibits the expression of key proteins in glycolysis to inhibit glycolysis level, thereby inhibiting cell migration and proliferation. Therefore, lncRNA ZNF674-AS1 could be a potent therapeutic target or a novel diagnostic molecule for patients suffering from liver cancer.

## 1. Introduction

Hepatocellular carcinoma (HCC) is one of the most prevalent malignant tumors with high mortality and morbidity. The incidence of HCC is increasing in recent years [1]. Despite some progress in the diagnosis and treatment of liver cancer over recent decades, patients still have a high recurrence rate and drug resistance after treatment, resulting in a poor prognosis [2, 3]. With the rapid development of molecular epidemiology and molecular biology, critical understanding has been accomplished in the pathogenesis of liver cancer. However, due to the high malignant degree of hepatocellular carcinoma, most cases have missed the best chance for surgery when patients visit a doctor, and even if radical resection is performed, the postoperative recurrence rate is high [4]. Hence, it is of great importance to find out the causes of the occurrence/advancement of hepatocellular carcinoma and seeking novel strategies for treatment.

Long noncoding RNA (lncRNA) is a category of RNA molecules containing a transcript length of over 200 nt. Rather than encode proteins, lncRNAs are capable of regulating the expression of genes at diverse levels, for instance, transcriptional and posttranscriptional regulations and epigenetic regulation [5, 6]. At present, it has been found that the functions of lncRNAs involve the majority of biological procedures for pathology and physiology of organisms. They are not only capable of regulating physiological procedures, for instance, cell proliferation, differentiation, and metabolisms, but also participate in the regulation of various pathological procedures of the body, including cancer and diabetes [7, 8]. In recent years, the most meticulous investigations on the function of lncRNA is its role in cancer [9]. A notable number of investigations have elucidated that lncRNA performs a critically essential task in the occurrence/advancement of tumors. It is used not only as a protooncogene to promote tumor formation but also as a tumor repressor gene to inhibit tumor cell proliferation and migration. Clinical and basic investigations have illustrated that the aberrant expression of lncRNA is strongly tied to the occurrence/advancement of diverse types of cancer. For example, knocking out lncRNA ANRIL in liver cancer cells significantly increases the expression levels of proapoptotic Bax and Bad and inhibits the antiapoptotic factors Bid and Bcl-2. These changes lead to slow cell growth and increased apoptosis [10]. In prostate cancer, LINC00115 inhibits cell invasion and proliferation by targeting the miR-212-5p/FZD5/Wnt/*β*-catenin axis [11]. Furthermore, lncRNA NEAT1 impedes gastric cancer cell invasion and proliferation and augments apoptosis through the regulation of the miR-500a-3p/XBP-1 axis [12].

lncRNA ZNF674-AS1 is a novel category of functional lncRNA discovered over recent decades and performs a substantial regulatory task in tumor cells. Previous studies have demonstrated that lncRNA ZNF674-AS1 is underexpressed in nonsmall cell lung cancer. Overexpression of lncRNA ZNF674-AS1 hinders cell proliferation and cycle arrest [13, 14]. The lncRNA ZNF674-AS1 can also inhibit the migration, invasion, and epithelial-mesenchymal transition of thyroid cancer cells through regulating the miR-181a/SOCS4 axis [15]. The role of lncRNA ZNF674-AS1 in the occurrence/advancement of hepatocellular carcinoma has not been presented. The primary objective of this research is to analyze the expression and function of lncRNA ZNF674-AS1 in hepatocellular carcinoma and explore the roles and mechanisms of lncRNA ZNF674-AS1 in the occurrence/advancement of hepatocellular carcinoma, in order to find new treatment target for hepatocellular carcinoma.

## 2. Materials and Methods

### 2.1. Tissue Specimen Collection

Twenty-five cases of liver cancer tissues and control paracancerous tissues were accumulated from Lu'an Hospital affiliated to Anhui Medical University. After surgical resection, the specimens were instantly frozen in liquid nitrogen and kept in a −80°C freezer. All patients' specimens were determined as primary liver cancer and were treated by surgery. At the same time, the clinical outcomes and different clinicopathological indicators of the registered cases were collected (Table 1). The contributors or their families who obtained specimens in the current research have signed an inform consent, and the procedures of this experiment have been confirmed through the Ethics Committee of Lu'an Hospital affiliated to Anhui Medical University.

### 2.2. Cell Culture

Human normal hepatocytes (THLE-2) and the cell lines of liver cancer (Hub7, SNU-182, SMMC-7721, HCC-LM3, and HepG2) were procured from Shanghai Cell Bank Center, Chinese Academy of Sciences. HepG2 and Huh7 cell lines were cultured under 37°C, in an incubator containing 5% CO_2_ and DMEM supplementing with 10% FBS and 1% streptomycin and penicillin. Cells were used for experiment when they reach more than 80% confluence.

### 2.3. Fluorescence Quantitative PCR Detection

The extraction of total RNA was achieved from cell lines and tissues employing the TRIzol method (in accordance with the guidelines). The reverse transcription of RNA into cDNA was accomplished in compliance with the guidelines of the PrimeScript RT reagent kit with gDNA Eraser. Add cDNA, related gene primers, and SYBR Green qPCR Master Mix, dilute the volume to 20 *μ*L with DEPC water, mix well, and perform detection on a fluorescence quantitative PCR instrument. The GAPDH was used as an internal reference control for the comparative level of gene expression and is evaluated according to the 2^−ΔΔCt^ method. The lncRNA ZNF674-AS1 and GAPDH primer sequences are referenced [13].

### 2.4. Transfection

The full-length sequence of lncRNA ZNF674-AS1 was obtained by the PCR amplification method, and it was connected to the expression vector pCDH. The recombinant plasmid was transferred into DH5*α* competent cells. The positive clones were selected and plasmids were extracted, digested, identified, and sequenced [14]. After the sequencing result was correct, the extracted plasmid, high expression vector, and packaging vector were cotransfected into HEK-293T cells, the virus supernatant was accumulated, and the virus was concentrated by PEG-it precipitation. The successfully packaged overexpressed lentiviral vector lncRNA ZNF674-AS1 and blank control group (vector) were infected with liver cancer cells accordingly, and the transfection effectiveness was discerned through fluorescence quantitative PCR.

### 2.5. CCK-8 Assay for Cell Viability

The cells of each group were seeded in the plates containing 96 wells and cultivated during the night hours. The medium was eliminated, and the CCK-8 test solution was added after rinsing with PBS and incubated for 2 hours at 37°C and 5% CO_2_. The absorbance at 450 nm of each group of cells at 0 h, 24 h, 48 h, and 72 h was measured in a microplate reader to reflect the changes in cell proliferation ability.

### 2.6. Colony Formation Assay

The phase of logarithmic growth was obtained for the cells of each group, then digested with 0.25% trypsin and pipetted into single cells, and seeded in a 6-well plate with 700 cells/well in a 37°C incubator with a CO_2_ content of 5%. The medium was improved every three days until following culturing for 21 days, rinsed 3 times with phosphate (PBS) buffer. Add 4% formaldehyde, fixed for 10 min, and rinsed 3 times with phosphate (PBS) buffer. 1 ml of 1% crystal violet was added for staining for 15 min, rinsed 3 times by utilizing phosphate (PBS) buffer, photographed, and observed the number of colonies formed.

### 2.7. Transwell Chamber Invasion Assay

Well-grown cells were taken, digested, and counted. Matrigel (BD company) was covered on the transwell chamber; the upper chamber of the transwell chamber was seeded with 200 *μ*L of serum-free medium comprising 1 × 10^5^ hepatoma cells; 500 *μ*L of DMEM complete medium comprising 10% fetal bovine serum was used as chemotaxis. The chemical factor was placed in the lower chamber and incubated for 2 days in a 5% CO_2_ incubator at 37°C; 4% paraformaldehyde was used for fixation for 30 min. Cells were stained with 1% crystal violet for 10 min. 5 high-power fields were randomly chosen for counting under an upright optical microscope, and the cells were counted. The mean value was used to represent the number of invasive cells.

### 2.8. Detecting Glucose and Lactate

The cell cultivation supernatants were collected, diluted, and added to 96-well plates. The standard wells were set at the same time, and then, the working solution was added. After 20 min of reaction at 37°C, the value of optical density (OD) for each well was evaluated at a wavelength of 570 nm, and a benchmark diagram was plotted for the assessment of the glucose and lactate content of liver cancer cells.

### 2.9. Intracellular ATP Detection

The ATP assay kit was employed to discern changes in the intracellular ATP content. The transfected cells were inoculated into a plate containing 24 wells and cultivated for 24 hours. 50 *μ*l of lysis solution was added to each well to lyse the cells, and the lysis supernatant of the cells was collected and discerned and analyzed with a chemiluminescence instrument.

### 2.10. Western Blot

The cells were lysed with lysate, the supernatant was accumulated by centrifugation, and the concentration of the protein was detected by the BCA technique [16]. Add 5  ×  loading buffer, denature the protein at 100°C for 5 min, and separate the protein by 12% SDS-PAGE. The loading volume of each well is 20 *μ*L, concentrating gel electrophoresis at 70 V for 40 min and separating gel electrophoresis at 110 V for 70 min. Subsequently, it was transferred to a PVDF membrane for 90 min with a current of 300 mA. By employing 5% BSA, the blotting membrane was blocked at ambient temperature for 2 hours, and next, the incubation was carried out by implementing the related primary antibody during the night hours at 4°C. Consequently, the blotting membrane were rinsed twice and incubated with the secondary antibody at ambient temperature for 2 hours. Following rinsing for two times, the chemiluminescence was detected with ECL substrate.

### 2.11. Statistical Methods

SPSS 16.0 computer program (SPSS Inc., USA) was employed for analyzing the outcomes of each group. The results were given as mean ± SD. GraphPad Prism 6.0 was implemented for graphing. The values of 0.05 was regarded a statistically meaningful discrepancy.

## 3. Result

### 3.1. LncRNA ZNF674-AS1 Is Expressed at Low Level in Liver Cancer Tissues and Cell Lines

**Figure 1:**
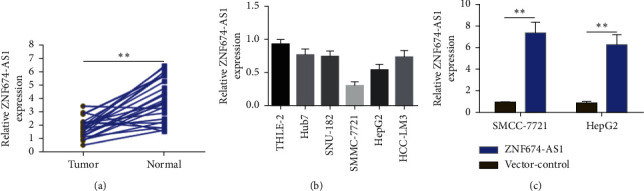
LncRNA ZNF674-AS1 is insufficiently expressed in liver cancer tissues and cell lines. Real-time PCR was employed for detecting the expression level of lncRNA ZNF674-AS1 in liver cancer tissues (a) and cell lines (b). (c) Established overexpression of lncRNA ZNF674-AS1 liver cancer cell line, ^∗∗^*P* < 0.01.

The expressions of lncRNA ZNF674-AS1 in liver cancer tissues and cells were discerned through fluorescence quantitative PCR. It was found that the expressions of lncRNA ZNF674-AS1 was substantially diminished in liver cancer tissues in comparison to the paracancerous tissues (Figure 1(a)). Cell experiments demonstrated that compared with human normal hepatocytes (THLE-2), the expression of lncRNA ZNF674-AS1 in hepatoma cell lines (Hub7, SNU-182, SMMC-7721, HepG2, and HCC-LM3) was significantly reduced (Figure 1(b)). The abovementioned results illustrate that abnormal expression of lncRNA ZNF674-AS1 can be relevant to the malignant biological behavior of liver cancer. Because of the low expression level of lncRNA ZNF674-AS1 in SMMC-7721 and HepG2 cells, an overexpression vector was constructed, and real-time quantitative PCR confirmed the overexpression of ZNF674-AS1 in cells transfected with the overexpression vector (ZNF674-AS1) in comparison to the blank control group (vector). The expression for lncRNA ZNF674-AS1 was substantially increased in SMMC-7721 and HepG2 cells (Figure 1(c)).

### 3.2. Overexpression of lncRNA ZNF674-AS1 Inhibits the Proliferation of Hepatocellular Carcinoma Cells

**Figure 2:**
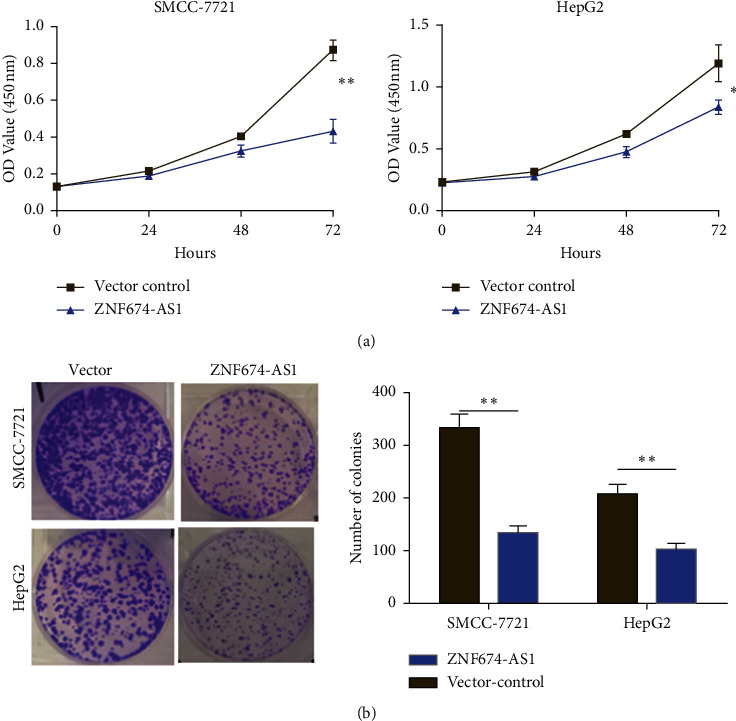
Overexpression of lncRNA ZNF674-AS1 hindered the proliferation of HCC cells. (a) CCK-8 assay for detecting the influence of overexpression of lncRNA ZNF674-AS1 on the proliferation of HCC cells. (b) Plate colony formation assay overexpression of lncRNA ZNF674, the influence of AS1 on colony formation of HCC cells, ^∗^*P* < 0.05, ^∗∗^*P* < 0.01.

The CCK-8 assay was applied to discern the influence of overexpression of lncRNA ZNF674-AS1 on the proliferation of liver cancer cells. The results illustrated that in comparison to the control group (vector), overexpression of lncRNA ZNF674-AS1 is able to substantially hinder the proliferation of liver cancer SMMC-7721 and HepG2 cells. Among them, the OD value decreased most significantly after 72 h (Figure 2(a)). The findings of the colony formation assay illustrated that compared with the blank control (vector) group, overexpression of lncRNA ZNF674-AS1 notably hindered the colony formation of SMMC-7721 and HepG2 cells (Figure 2(b)). These results illustrated that overexpression of lncRNA ZNF674-AS1 is able to hinder the proliferation of HCC cells.

### 3.3. Overexpression of lncRNA ZNF674-AS1 Impedes the Migration and Invasion of HCC Cells

**Figure 3:**
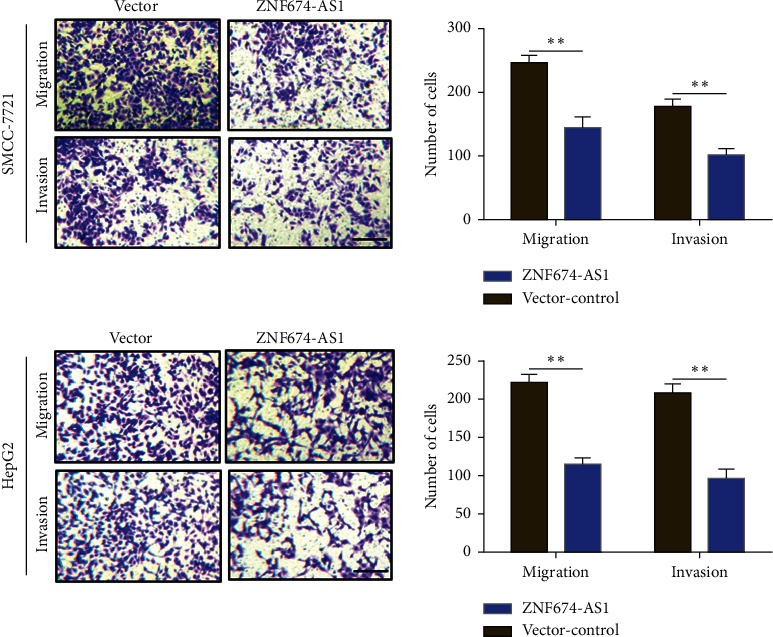
Overexpression of lncRNA ZNF674-AS1 impedes the migration and invasion of hepatoma SMMC-7721 and HepG2 cells, ^∗∗^*P* < 0.01. Scale bar = 100 *μ*m.

The effect of overexpression of lncRNA ZNF674-AS1 on the migration and invasion ability of HCC cells was appraised. Transwell invasion and migration assay showed that compared with the blank control (vector), overexpression of lncRNA ZNF674-AS1 substantially impeded the migration and invasion capability of SMMC-7721 and HepG2 cells (Figure 3). These results demonstrated that overexpression of lncRNA ZNF674-AS1 is capable of hindering the migration and invasion of HCC cells.

### 3.4. Overexpression of lncRNA ZNF674-AS1 Inhibits the Level of Aerobic Glycolysis in HCC Cells

**Figure 4:**
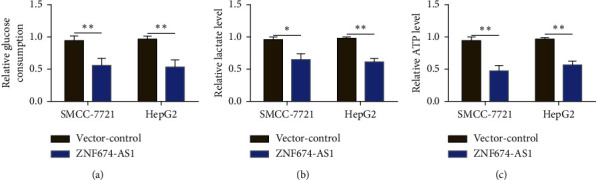
Influence of overexpression of lncRNA ZNF674-AS1 on the level of aerobic glycolysis in SMMC-7721 and HepG2 cells. Effects of overexpression of lncRNA ZNF674-AS1 on the glucose metabolism (a), intracellular lactate generation (b), and ATP production (c) in liver cancer HepG2 and SMMC-7721 cells, ^∗^*P* < 0.05, ^∗∗^*P* < 0.01.

The enhanced aerobic glycolytic capacity of tumors is an essential mechanism for the rapid proliferation and invasion of tumors. To investigate the specific mechanism of the influence of lncRNA ZNF674-AS1 on the biological functions of liver cancer cells, the aerobic glycolytic activity was detected. The results illustrated that in comparison to the blank control (vector), overexpression of lncRNA ZNF674-AS1 is able to hinder the level of glucose adsorption in liver cancer SMMC-7721 and HepG2 cells (Figure 4(a)), while reducing intracellular lactate (Figure 4(b)) and the production of adenosine triphosphate (ATP) (Figure 4(c)) indicated that lncRNA ZNF674-AS1 performs a substantial task in maintaining the level of aerobic glycolysis in HCC cells.

### 3.5. Effects of Overexpression of lncRNA ZNF674-AS1 on the Expression of Aerobic Glycolysis-Related Proteins in Hepatocellular Carcinoma Cells

**Figure 5:**
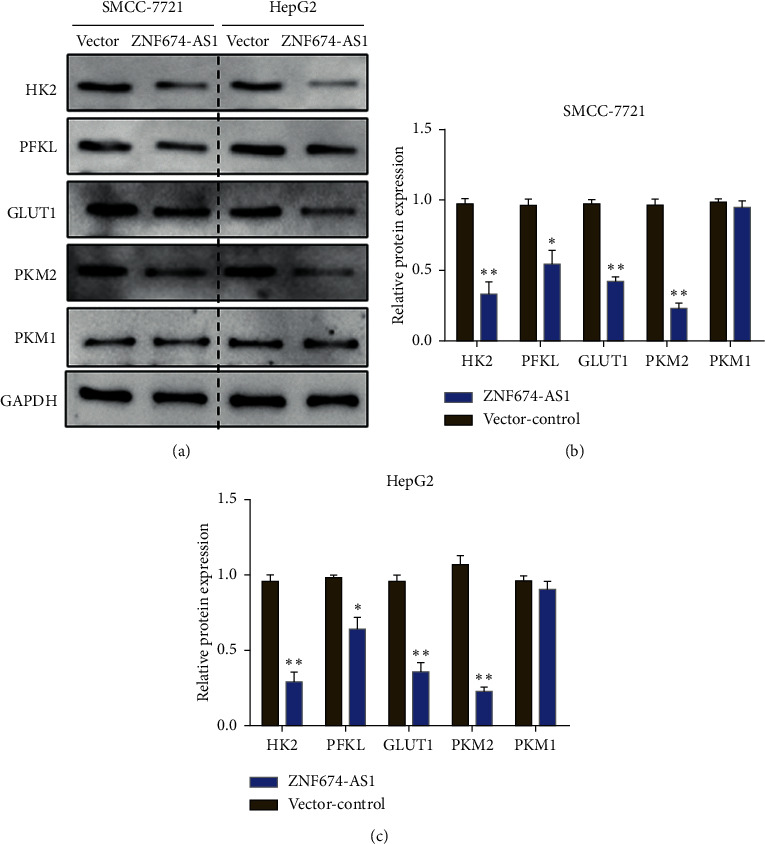
Western blot detection of the impact of overexpression of lncRNA ZNF674-AS1 on the expression of aerobic glycolysis-related proteins in liver cancer HepG2 and SMMC-7721 cells, ^∗^*P* < 0.05, ^∗∗^*P* < 0.01.

The above results illustrated that overexpression of lncRNA ZNF674-AS1 inhibits the level of aerobic glycolysis in liver cancer cells. In order to find out the role of lncRNA ZNF674-AS1 in affecting aerobic glycolysis, Western blot was executed to evaluate the effect of overexpression of lncRNA ZNF674-AS1 on liver cancer and the effects of cellular aerobic glycolysis-related protein expression. The findings illuminated (Figure 5(a)) that compared with the blank control (vector), overexpression of lncRNA ZNF674-AS1 inhibited the expression of key proteins HK2, PFKL, PKM2, and GLUT1 in the glycolysis process of liver cancer SMMC-7721 and HepG2 cells. There was no meaningful impact on the expression of PKM1 (Figures 5(b) and 5(c)).

## 4. Conclusion

Cancer cells have the ability to proliferate indefinitely and can affect other organs and tissues through invasion and metastasis [17]. Glycolysis can not only provide energy for normal cells but also an energy source for cancer cells, and the products in the process of glycolysis are beneficial to the proliferation of cancer cells [18]. Previous investigations have illustrated that enhanced glycolysis is a way for cancer cells to adapt to the hypoxic environment. While providing energy for cancer cells, a large amount of lactic acid is produced to acidify the extracellular matrix, which is beneficial to the invasion and metastasis of cancer cells [19, 20]. Hence, the process of glycolysis is one of the important drivers of malignant transformation of tumors. Recent studies have shown that lncRNA performs a substantial task in the regulation of tumor cell glycolysis. For example, in liver cancer cells, lncRNA LNCAROD increases the expression level of PKM2 through miR-145-5p and increases cellular aerobic glycolysis to promote cancer development [21]. The lncRNA FIRRE promotes liver cancer cell proliferation and glycolysis through improving CREB-mediated transcription as well as PFKFB4 expression [22]. Therefore, identifying novel lncRNAs that fulfill pivotal tasks in the glycolysis process of liver cancer and elucidating their regulatory mechanisms are essential for the timely determination and targeted treatment of liver cancer. The outcomes of the current survey show that lncRNA ZNF674-AS1 is inadequately expressed in liver cancer cell lines and tissues. Functional experiments show that overexpression of lncRNA ZNF674-AS1 is capable to hinder the invasion and proliferation of liver cancer cells and reduce the glycolytic capacity of liver cancer cells. The above experiments show that lncRNA ZNF674-AS1 affects the proliferation and invasion ability of liver cancer cells with the aid of regulating the glycolysis process of liver cancer cells.

Recently, functional genomics investigations have divulged that a notable quantity of long noncoding RNAs (lncRNAs), which have been neglected in cells for a long time, can behave as predominant regulators of gene expression through diverse strategies, including transcriptional regulation, translation, and protein modification. The lncRNAs fulfill pivotal roles in diverse disease processes [9]. For example, lncRNA-D16366 is substantially reduced in serum and tissue of liver cancer, and its expression is associated with portal vein tumor thrombus, tumor size, treatment, and tumor metastasis and is an independent diagnostic and prognostic indicator for liver cancer [23]. The lncRNA SUMO1P3 serves as an advantageous predictor of liver cancer prognosis and a potent therapeutic target [24]. In addition, lncRNAs also accomplish a predominant task in the proliferation, metastasis, and invasion of liver cancer. For example, lncRNA TUG1 is considerably expressed in hepatoma cells and augments the invasion and migration of hepatocellular carcinoma through targeting the miR-137/AKT2 axis [25]. The outcomes of the current survey illuminated that lncRNA ZNF674-AS1 was expressed at low level in liver cancer cells and tissues, and overexpression of lncRNA ZNF674-AS1 in liver cancer cells could substantially inhibit the proliferation, plate colony formation, migration, and invasion of liver cancer cells. The achievements of this exploration are consistent with other investigations that lncRNA ZNF674-AS1 is underexpressed in thyroid cancer, and overexpression of lncRNA ZNF674-AS1 can remarkably inhibit cell proliferation, migration, invasion, and epithelial-mesenchymal transition (EMT) [15]. In addition, lncRNA ZNF674-AS1 was downregulated in the tissues and cells of nonsmall cell lung cancer and could substantially hinder cell invasion and migration [14].

Cancer cells possess special functions of the glucose metabolism. They perform active glycolysis even in the presence of adequate oxygen which is even its main energy source [26, 27]. In liver cancer cells, aerobic glycolysis is a hallmark of liver cancer cells that is responsible for regulating liver cancer proliferation, invasion, metastasis, immune evasion, drug resistance, and cell stemness [28].

Histone deacetylase 11 (HDAC11) is substantially expressed in liver cancer and is strongly tied to the prognosis of the disease. HDAC11 knockdown promotes histone acetylation in the related promoter region to increase the transcription of LKB1, consequently activating the signaling pathway of AMPK and hindering the glucose glycolytic pathway, which accordingly inhibits cancer stemness and liver cancer progression and increases sensitivity to sorafenib [29]. The lncRNA SLC2A1-AS1 is able to regulate aerobic glycolysis and development of hepatocellular carcinoma by inhibiting the STAT3/FOXM1/GLUT1 signaling pathway [30]. In addition, previous studies found that lncRNA ZNF674-AS1 can regulate granulosa cell glycolysis and proliferation through interactions with ALDOA [31], suggesting that lncRNA ZNF674-AS1 can take part in the regulation of intracellular glycolysis. Our study demonstrated that overexpression of lncRNA ZNF674-AS1 can remarkably hinder the consumption of glucose and diminish the levels of intracellular lactate and ATP in liver cancer cells. Studies have shown that there are three rate-restricting enzymes in the glycolytic procedure, comprising phosphofructokinase-1 (PFK1), hexokinase-2 (HK2), and M2-type pyruvate kinase (PKM2). These enzymes regulate aerobic glycolysis in liver cancer cells and can be regulated in several ways [32–34]. In addition, glucose transporter-1 (GLUT1) and phosphofructokinase-1 (phosphofructokinase-1, liver type, PFKL) are pivotal in the glycolysis of tumor cells [35, 36]. Herein, it was demonstrated that overexpression of lncRNA ZNF674-AS1 could remarkably inhibit the expression of PFKL, HK2, GLUT1, and PKM2 in the glycolysis metabolism of liver cancer cells. However, it is unclear whether lncRNA ZNF674-AS1 regulates the molecules of glycolysis in liver cancer cells.

Taken together, the present research illustrates that lncRNA ZNF674-AS1 is inadequately expressed in liver cancer cells and tissues. Overexpression of lncRNA ZNF674-AS1 is able to hinder liver cancer cell proliferation, plate colony formation, cell invasion, and migration ability. Moreover, overexpression of lncRNA ZNF674-AS1 reduces the expression of HK2, PFKL, PKM2, and GLUT1 and inhibits glycolysis of liver cancer cells. In the follow-up investigation, our study provides a new direction for the treatment of liver cancer.

## Figures and Tables

**Table 1:** Clinical baseline information of the patient.

Features	*n*
Gender	
Male	15
Female	10

Age	
<50	4
≥50	21

HBV infection	
Yes	16
No	9

Diameter of tumor (cm)	
<5	9
≥5	16

Differentiated degree	
Well differentiated	9
Moderately differentiated	12
Poorly differentiated	4

TNM stage	
T1+T2	13
T3+T4	12

BCLC stage	
A	3
B	9
C	11
D	2

Microvascular invasion	
Yes	2
No	23

Primary site	
Peripheral	15
Intermediate type	10

Number of primary lesions	
Single	24
Multiple	1

## Data Availability

The data used to support this study are available from the corresponding author upon request.
